# Biochemical Consequences of a Leucine-to-Cysteine Clamp Substitution in Lipoxygenases

**DOI:** 10.3390/biom15081153

**Published:** 2025-08-11

**Authors:** Samuel G. Hill, Katherine DeFeo, Adam R. Offenbacher

**Affiliations:** Department of Chemistry, East Carolina University, Greenville, NC 27858, USA; hillsa19@ecualumni.ecu.edu (S.G.H.); defeok22@students.ecu.edu (K.D.)

**Keywords:** lipoxygenase, manganese, enzyme kinetics, kinetic isotope effects, fatty acids

## Abstract

Lipoxygenases (LOXs) are a family of metalloenzymes that oxidize polyunsaturated fatty acids producing cell-signaling hydroperoxides. Fungal LOXs have drawn interest because of their roles in plant and animal pathogenesis. A new subfamily of annotated fungal LOXs has been predicted. One of its unique structural features is the presence of a cysteine amino acid encoded at the invariant leucine clamp. Herein, we isolate three representatives of this LOX subfamily from recombinant expressions in both yeast and bacterial cultures. Metal analysis indicates that the proteins accommodate a mononuclear manganese ion center, similar to other eukaryotic LOXs, but have nominal LOX activity. The functional consequence of the non-conservative mutation is further explored using a Leu-to-Cys (L546C) variant of soybean lipoxygenase, a model plant orthologue. While this L546C variant has comparable structural integrity and metal content to the native enzyme, the variant is associated with a 50-fold decrease in the first-order rate constant. The presence of cysteine at 546, compared to leucine, alanine, or serine, also results in a distinctive kinetic lag phase and product inhibition. The collective data highlight that Cys encoded at the Leu clamp is detrimental to LOX activity. Potential biological functions of these annotated fungal LOXs are discussed.

## 1. Introduction

**Scheme 1:**
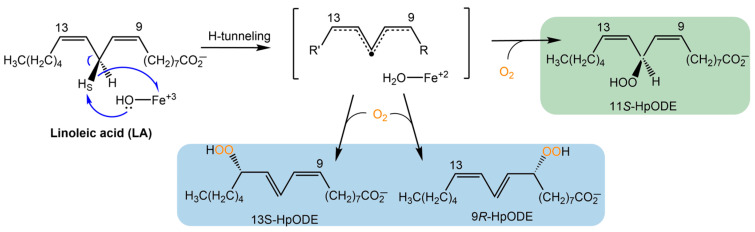
LOX reaction mechanism. The LOX reaction is initiated by a rate-limiting C-H bond cleavage followed by insertion of molecular oxygen. Products from plant and animal LOX are highlighted in blue. The initial products of fungal LOXs are highlighted in green; these products can undergo β-fragmentation to produce 9- and 13-hydroperoxides [3,6,11].

Lipoxygenases (LOXs) are a diverse family of enzymes that oxidize polyunsaturated fatty acids (Scheme 1). Bioactive hydroperoxide products stemming from the canonical LOXs of plants and animals play important roles in growth and development, cell-signaling, and inflammatory responses [1,2,3]. Fungal LOXs were discovered nearly 30 years ago [4]. The first fungal LOX to be isolated and characterized was from the take-all wheat fungus, *Gaeumannomyces graminis*, which contains a single LOX gene encoding the enzyme GgLox. Studies from this enzyme and others revealed key differences from plant and animal orthologues.

GgLox, isolated from its native host, showed an anomalous migration in SDS-PAGE analysis that corresponded to the protein being decorated with multiple post-translationally modified *N*-linked glycans at the protein surface. GgLox was subsequently cloned and isolated from *Pichia pastoris* cultures [5]. The recombinant enzyme displayed similar activity and retained the same *N*-linked glycan patterns to the native enzyme isolated from the host organism. Since then, other fungal LOXs have been isolated from recombinant expressions using either yeast (*P. pastoris*) or bacteria (*Escherichia coli*) [3,6,7].

Fungal LOXs have been isolated with a catalytically active, mononuclear manganese cofactor, whereas the catalytic cofactor in canonical plant and animal LOXs is a mononuclear, non-heme iron ion [3,8,9]. The metallocentre is essential for initiating catalysis, via a rate-limiting C-H cleavage by hydrogen tunneling [10], followed by molecular oxygen insertion to produce the hydroperoxide product (Scheme 1). Fungal LOXs have been shown to produce a unique bis-allylic hydroperoxide, such as (9Z,11S,12Z)-11-hydroperoxyoctadeca-9,12-dienoic acid (11*S*-HpODE) from the oxidation of linoleic acid (LA) (Scheme 1) [6,11]. Plant and animal LOXs produce only conjugated hydroperoxides (e.g., 9 or 13). Fungal enzymes can further isomerize 11*S*-HpODE to form 9- and 13-hydroperoxides. However, a distinction is that oxygen insertion by fungal LOXs occurs in a suprafacial manner, whereas other eukaryotic LOXs perform oxygen insertion antarafacially [3].

**Figure 1:**
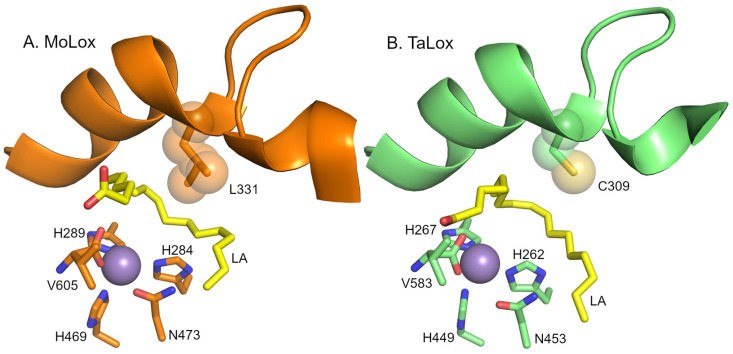
Models of the active sites of fungal LOXs. Panel (**A**) presents the model from the X-ray crystal structure of MoLox (PDB: 5FNO [17]). Panel (**B**) presents an AlphaFold3 prediction model of TaLox. The fatty acid (LA) is modeled and depicted as yellow sticks. The metal center (modeled as Mn^2+^) is shown as a purple sphere. The Leu (or Cys) clamp is represented as spheres for reference of volume. The water/hydroxide molecule attached to the metal was omitted for clarity.

Of the characterized manganese LOXs, five of them originate from some of the most devastating plant-pathogenic fungi [3], including *G. graminis* (take-all wheat fungus, with a single lipoxygenase, GgLox) and *Magnaporthe oryzae* (rice blast fungus, with a single lipoxygenase, MoLox). A bioinformatics study identified a total of 48 predicted fungal LOX sequences, including the five previously biochemically and/or structurally characterized enzymes [12]. These predicted LOX gene sequences were divided into three subfamilies based on phylogenetic analysis. The fungal LOXs studied to date, including GgLox and MoLox, are all classified as prototypical fungal LOXs (or referred to as class I herein). A second subfamily of predicted fungal LOXs, designated herein as class II, is found primarily from organisms that are pathogens of parasites, with a few members originating from plant pathogens and fungi used for bio control [12]. One key feature that distinguishes these putative class II isozymes from the prototypical class I fungal LOXs is the presence of a cysteine residue at the position of the invariant Leu clamp residue (Figure 1).

The Leu clamp is completely conserved in class I fungal and all canonical Fe-LOXs [13]. The leucine residue does not bind metal but is instead a cornerstone residue in positioning the pentadiene framework of substrate with respect to the catalytically essential metal cofactor for effective hydrogen abstraction to initiate enzyme catalysis [13,14]. Previous site-directed mutagenesis studies focusing on the leucine clamp residue in the model plant LOX from soybean (SLO; residue L546), a human LOX (15-LOX-2; residue L420) and MoLox (residue L331) revealed detrimental impacts on enzyme rate when the Leu was mutated to the volume reducing sidechain, alanine (ca. 20- to 100-fold decreases in enzyme rate) [9,15,16]. Thus, the natural, yet non-conservative substitution of this Leu clamp residue in the predicted class II fungal LOXs may be of functional interest.

In this report, we have recombinantly expressed and isolated three of the nine predicted class II fungal LOXs using yeast and/or bacterial cultures. In vitro kinetic assays show poor-to-no LOX activity, while the structural studies suggest that the proteins properly adopt the LOX fold. In addition, we examined the kinetic consequences of substituting cysteine for the leucine clamp in soybean lipoxygenase, a well-studied plant LOX isoenzyme. The collective information helps us to further understand the importance of maintaining a bulky aliphatic sidechain at the Leu clamp positioin for effective LOX catalysis.

## 2. Materials and Methods

### 2.1. General Materials

Buffers and media for protein expression were obtained from Sigma (St. Louis, MO, USA), Thermo Fisher (Waltham, MA, USA), or VWR (Radnor, PA, USA) at ACS grade or higher. All buffers were prepared with ultrapure water using the Elga water purification system (Elga LabWater, High Wycombe, UK). Fatty acid substrates were purchased from Cayman (Cayman Chemicals, Ann Arbor, MI, USA).

### 2.2. Selection of Class II Fungal LOXs

There are nine predicted class II fungal LOXs (each annotated as a LOX in the NCBI) [12]. From this list, we selected three representatives in this work for biochemical studies: 1) *Trichoderma arundinaceum* (TaLox) (organism function: bio control of *B. cinerea*), *Periconia macrospinosa* (PmLox) (root-fungus symbiosis), and *Cordyceps javanica* (CjLox) (control of Asian citrus jumping plant lice). *T. arundinaceum* is a candidate for further studies due to closely related sequences in the other *Trichoderma* species [18]. *P. macrospinosa* is a mychorriza fungus and thus plays a role in root-fungus symbiosis [19]. *C. javanica* is a pathogen of insects [20]. While all three of the putative class II LOXs have a cysteine encoded at the invariant Leu clamp site, there are other sequence variations, including the C-terminal peptide, with the carboxyl terminus serving as a ligand to the catalytically essential metal, and the active site ‘Bo1’ determinant that may influence the positioning of substrate for oxygen insertion [21]. In addition, these three representatives were selected in part on differing number of predicted *N*-linked glycosylation sites (see Table S1).

The protein sequences of CjLox, PmLox, and TaLox are listed in Table S2. The genes were synthesized to optimize the codon sequences for expression in *P. pastoris*. Similar to GgLox and MoLox, each of the class II fungal LOX genes encode an 18–20 amino acid sequence at their N-termini that are predicted as secretion peptides. The signal peptides were predicted using the SignalP 6.0 server [22]. The signal sequence peptides were removed prior to insertion into the expression vector (see below).

### 2.3. Expression and Purification of MoLox and Class II Enzymes from Yeast Cultures

MoLox was expressed in and isolated from the *Pichia pastoris* X-33 yeast strain, as previously described [9,23,24]. Using this strategy, the genes for CjLox, PmLox, and TaLox genes were synthesized by Genscript (Genscript, Piscataway, NJ, USA) and subcloned into the pPICZa plasmid, in line with the α secretion peptide. The plasmids were linearized by SacI restriction enzyme and transformed into *P. pastoris* X33 using the lithium chloride heat shock method. Transformed cells were plated on YPD (yeast extract/peptone/dextrose) plates containing Zeocin as the antibiotic. The plates were incubated at 28–30 °C for 3 days or until colonies appeared. A single colony of these stable cell lines was selected and grown in a starter culture containing BMGY minimal medium (phosphate-buffered yeast nitrogen bases with glycerol) until desired growth was reached. For large scale expression (4 L), a starter culture (~200 mL) was grown to an OD_600_ (optical density at 600 nm) of 20. The cells were collected by centrifugation, washed in water (to remove glycerol), and resuspended in expression minimal medium of BMMY (phosphate-buffered yeast nitrogen bases with methanol) in baffled Thompson flasks. Methanol was added daily (0.5% final concentration) to induce protein expression. The protein expressions were typically conducted for 3–4 days in shaking incubators (30 °C).

Because the fungal LOX gene is expressed in line with the α secretion signaling peptide, class II LOXs were secreted from the yeast into the media. The cultures were centrifuged to remove the cells. The supernatant was collected and neutralized to pH 7.0; ammonium sulfate was subsequently added as a solid to a 1 M concentration. The class II LOX protein was purified using hydrophobic interaction chromatography (Phenyl Sepharose). After the protein was loaded onto the Phenyl Sepharose medium, the column was washed with ~5 column volumes of high salt buffer (1 M ammonium sulfate and 25 mM potassium phosphate, pH 7). The protein was eluted using a linear salt gradient: 1 M ammonium sulfate and 25 mM potassium phosphate (high salt) and 25 mM potassium phosphate with no ammonium sulfate (low salt). Fractions that displayed enzyme activity and/or showed protein absorbance at A280 were concentrated using a JumboSep concentrator (30 kDa MWCO) and further purified using size exclusion chromatography (SEC) with a HiPrep S-200 column (equilibrated with 50 mM HEPES, 150 mM NaCl buffer, pH 7.5) on an ÄKTA FPLC. Note that fractions from the class II purifications had no notable activity; however, fractions were selected based on absorbance spectra consistent with protein and confirmed with SDS-PAGE. The purified protein was collected, concentrated to 0.1–0.2 mM, flash frozen in aliquots, and stored in the −80 °C freezer until further use.

### 2.4. Preparation of Soybean Lipoxygenase (SLO) Variants Mimicking Class II LOXs

The original pT7-7 plasmid [25] containing soybean lipoxygenase (SLO) gene was transformed into *E. coli* BL21 (DE3) Codon Plus RIL cells. Variants of SLO (L546C and L546S) were prepared using site directed mutagenesis with the Qiagen QuickChange kit. The mutation sequences were analyzed and confirmed by whole plasmid sequencing. SLO was expressed and purified as described in detail in reference [26]. In brief, after lysis of the harvested cell cultures, the protein was dialyzed against 20 mM bis-TRIS (pH 6.0) and purified by cation exchange chromatography (SP Sepharose and Uno-S columns). The purified protein, as determined by SDS-PAGE, was dialyzed against 0.1 M sodium borate buffer (pH 9.0), flash frozen, and stored at −80 °C until use. To measure free thiols, the Ellman’s test was carried out for the L546C variant with 5,5′-dithiobis-(2-nitrobenzoic) acid (DTNB) using standard protocols [27].

### 2.5. Expression and Purification of TaLox from E. coli

To express TaLox in *E. coli*, the gene was sub-cloned into a pET28a vector in line with an encoded N-terminal His_6_ tag. The plasmid was transformed into *E. coli* BL21 (DE3) Codon Plus RIL cells. An overnight starter culture was used to inoculate 2xYT media supplemented with 35 mg/L kanamycin. For preparing Mn-enriched TaLox from *E. coli*, the overnight starter culture was centrifuged and washed with water. These cells were added to a M9-based minimal media lacking iron and supplemented with manganese, according to our published protocol [26]. In brief, 900 mL of sterile M9 media was prepared in EDTA-washed polycarbonate Fernbach flasks. Each flask was also prepared with (final concentrations) 0.5% (*w*/*v*) glucose, 3 g/L casamino acids, 100 mg/L thiamine, 2 mM MgSO_4_, 0.1 mM CaCl_2_, 0.15 mM MnCl_2_, 35 mg/L kanamycin. The cells were grown in a shaking incubator at 37 °C to an OD_600_ of ~0.8, at which point the temperature was dropped to 17 °C. Then, 0.1 mM isopropyl β-D-thiogalactopyranoside (IPTG) was added to each flask for induction. After 48 h, the cells were harvested using centrifugation.

Both proteins were purified in the same manner. The cell pellet was resuspended in a lysis buffer (50 mM sodium phosphate, 100 mM NaCl, pH 8.0, 8% glycerol, and 2 mM magnesium sulfate supplemented with lysozyme, DNase I, and AEBSF). The cells were disrupted by sonication and cellular debris was removed by centrifugation. The supernatant was loaded onto a gravity fed Ni-NTA column. The column was washed with 15 column volumes of buffer A (20 mM Tris, 20 mM imidazole supplemented with 0.5 M NaCl, pH 8) to remove non-specifically bound protein from the column. The enzyme was eluted from the column using buffer B (buffer A with 200 mM imidazole). The fractions were analyzed for protein content using UV-vis and SDS-PAGE. The protein samples were concentrated using a 30 kDa MWCO concentrator and further purified by size exclusion chromatography using a Superdex 200 increase 10/300 GL column on an ÄKTA go fast-protein liquid chromatography system. The column was equilibrated with 50 mM HEPES (pH 7.5), 150 mM NaCl buffer. The purified proteins were aliquoted as necessary and stored in a −80 °C for future use.

### 2.6. Steady-State Kinetics

Steady-state kinetics of the fungal LOXs were measured using a Hansatech Liquid-Phase Oxygen Electrode and Oxygraph+ system. The Oxygraph+ system is equipped with an S1 oxygen electrode disc, which is coupled with an electrode control unit. The reactions were performed in 0.1 M sodium borate (pH 9.0) and at 25 °C as a function of substrate concentrations: 100, 50, 30, 15, 5, and 2 µM (final concentration) of linoleic acid, LA. Steady-state kinetics for SLO variants were measured using UV-vis spectroscopy. The reactions were recorded at a single wavelength (234 nm) kinetics mode to capture the absorbance of the conjugated hydroperoxide products. The reactions were performed in 0.1 M sodium borate buffer (pH 9.0) and at 30 °C as a function of substrate concentrations: 100, 50, 25, 20, 10, 5 μM LA. Deuterated linoleic (D_31_-LA) acid was used to determine the kinetic isotope effects. All kinetic data were collected at minimum in triplicate and analyzed using IGOR Pro (version 5.0). The data were plotted (rate versus substrate concentration), and the curves were fitted using the Michalis-Menten equation to obtain *k*_cat_ and K_m_. All kinetic parameters are reported as the average of three independent kinetic measurements ± standard error of the mean (s.e.m.).

### 2.7. Metal Analysis

LOX samples were analyzed for Mn, Fe, Cu, and Zn using Inductively Coupled Plasma Optical Emission Spectroscopy (ICP-OES). The LOX samples were prepared with an expected 300–500 ppb metal concentration range as follows: nitric acid was added (to 2% final concentration); the samples were centrifuged to remove any aggregate denatured protein, and the supernatant was transferred to a fresh tube. The ICP-OES method was created to detect copper (324, 327 nm), iron (238 and 260 nm), zinc (202 and 214 nm), and manganese (258 and 259 nm), with these wavelengths selected for the highest signal while avoiding spectral overlap. The raw data (intensity counts) were converted to ppb based on a standard calibration curve of seven standard concentrations, ranging from 0 (blank) to 1000 ppb. Ferrozine assays were also used to measure iron content in SLO variants [26].

### 2.8. DeepMind AlphaFold 3 Predictions of Protein Models

We constructed five structural predictions of MoLox and the three putative class II fungal LOXs. The confidence values consistently scored above 90% for all models. The crystal structure of MoLox and the AlphaFold 3 (AF3) model produced an RMSD value of 0.27 Å, which provided good agreement between the models. See Figure S1 for comparisons.

### 2.9. Circular Dichroism (CD) Spectroscopy

CD experiments for structural information were conducted on a Jasco J-815 CD Spectrometer. The LOX samples were prepared at concentrations in the range of 1.5–2 µM in 25 mM potassium phosphate (pH 7) buffer for fungal LOXs and 25 mM sodium borate (pH 9) buffer for SLO variants. CD spectra were collected at 20 °C in the range of 190–250 nm with a scan rate of 100 nm/min and a digital integration time (D.I.T.) of 4 s. The high tension (HT) voltage for these experiments were maintained at or below 600 mV.

### 2.10. Differential Scanning Calorimetry (DSC)

DSC experiments were conducted on a TA-instruments Nano-DSC microcalorimeter. The various LOX samples were prepared at 30–40 µM concentration in 50 mM HEPES (pH 7.5) with 0.15 M NaCl for the fungal LOXs or 0.1 M sodium borate (pH 9.0) for SLO. The DSC experiments were measured across the temperature range of 30–80 °C (heat only) at a rate of 1 °C min^−1^ with a constant pressure of 3 atm. The data was analyzed using TA Instruments NanoAnalyze software (version 3.12.5). Data were collected and reported from 2–3 samples.

## 3. Results

### 3.1. Isolation of Class II Fungal LOXs

**Figure 2:**
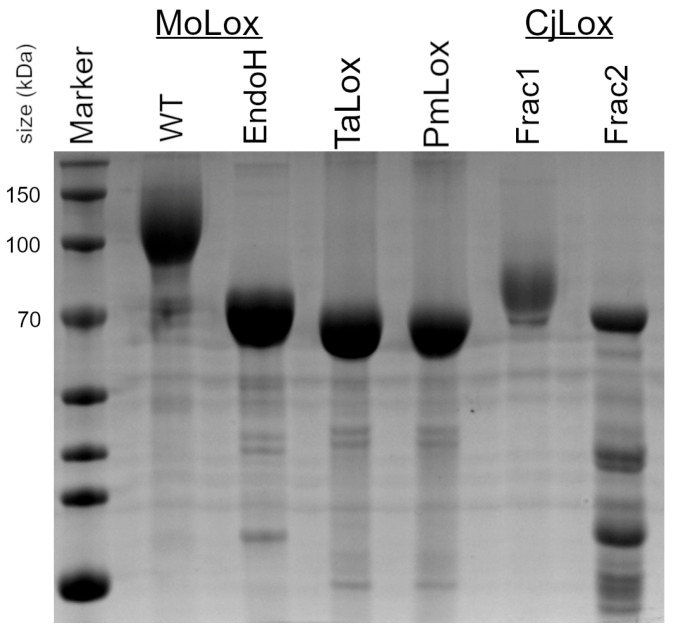
SDS-PAGE of fungal LOXs. EndoH is a sample of wild-type (WT) MoLox that has been treated with endoglycosidase H (EndoH) to remove the *N*-linked glycans [24].

Figure 2 presents the SDS-PAGE analysis of the predicted class II fungal LOX representatives that were isolated from *P. pastoris* yeast cultures in a similar manner to GgLox and MoLox. Both TaLox and PmLox were purified to near homogeneity. Based on the markers, the class II proteins migrated consistently with their theoretical masses (65–67 kDa; see Table S1). For comparison, the SDS-PAGE of MoLox is also presented in Figure 2. The theoretical molecular weight of MoLox is 67 kDa; however, the band migrates at a much higher mass of ~100–140 kDa [23]. The decreased gel mobility (i.e., increased apparent molecular weight) of MoLox in the SDS-PAGE is caused by the presence of post-translational *N*-linked glycans. In addition to the sluggish migration of the wild-type MoLox, there is considerable smearing of the band in the SDS-PAGE that is attributed to the heterogeneity of the glycan structure(s). MoLox contains eight predicted sites for *N*-linked glycans that have been corroborated from a combination of biochemical, mass spectrometry, and crystallography experiments [17,23,24]. Removal of the glycans by endoglycosidases, such as EndoH, results in a new migration pattern for MoLox that agrees with the predicted molecular weight of the non-glycosylated protein.

To date, class I fungal LOXs isolated from their native source or recombinantly from yeast cultures have several *N*-linked post-translational carbohydrates appended to their surface. Using the NetNGlyc server [28], the three representative class II LOXs are predicted to have either two (TaLox, CjLox) or seven (PmLox) *N*-linked glycans. Despite these predictions, both TaLox and PmLox migrate to their theoretical molecular weights. The migration of these proteins (as isolated from yeast cultures) is therefore consistent with no post-translational glycosylation. To further check for lack of *N*-linked glycosylation, SDS-PAGE was performed for a series of TaLox samples that were treated with either glycosidase, EndoH or PNGaseF (Figure S2). These TaLox samples migrated identically to TaLox isolated from the yeast culture. Thus, the result of the treatments further corroborated the conclusion that these class II LOXs are isolated from *P. pastoris* without *N*-linked glycosylation.

CjLox eluted from the SEC in two fractions. In the SDS-PAGE, the first fraction migrated at a higher molecular weight than its theoretical mass (Figure 2). We assign this fraction to CjLox with *N*-linked glycosylation. The second fraction migrated in the SDS-PAGE at ~70 kDa, consistent with its theoretical molecular weight. However, this latter sample had a few contaminants that co-eluted with CjLox. The first fraction was unstable based on differential scanning calorimetry results (see below). Further, the overall yields for either fraction of CjLox were considerably lower than either PmLox or TaLox. As a result, we focused on TaLox and PmLox.

### 3.2. Protein Folding and Stability

**Figure 3:**
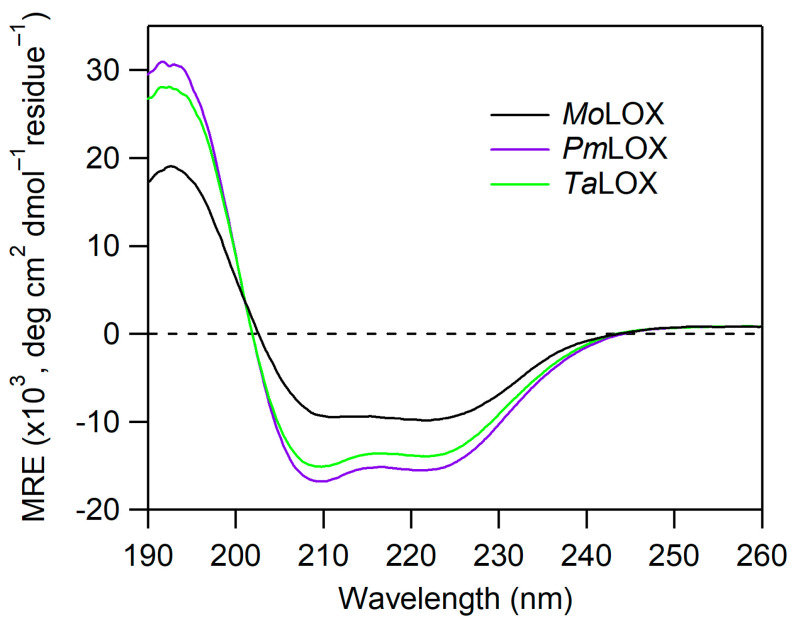
CD spectra of MoLox and class II fungal LOXs isolated from *P. pastoris* cultures. The CD spectra are normalized based on their protein concentrations and residue number (i.e., mean residue ellipticity, MRE). The buffer was 25 mM potassium phosphate (pH 7) and the temperature was 20 °C.

CD spectroscopy was used to analyze protein folding and secondary structures of the putative class II fungal proteins. The crystal structure of MoLox has been previously reported [17]. Unlike plant and animal LOXs, MoLox contains only the catalytic domain that is mostly α-helical. Based on homology to this structure of MoLox and their AlphaFold-3 prediction models, these class II LOXs are also expected to be primarily helical (Figure S1). The CD spectral overlays of MoLox, PmLox, and TaLox are shown in Figure 3. Their CD traces have two minimum peaks at ~222 and 208 nm and a positive feature at 193 nm, consistent with an expected α-helical structure. The CD data sets represent the mean residual ellipticity in which the raw ellipticity signal is corrected to protein concentration and amino acid length. Based on this comparison, the three proteins are similar, though there is some minor variation in the molar ellipticity between class II proteins and MoLox. To examine the structural information of class II LOXs further, we generated predicted CD spectra using PDBMD2CD [29] with the AF3 predicted models of PmLox and TaLox (Figure S3). Their experimental CD data agreed well with the predicted CD spectra using the AF3 predicted structural models. It is worth noting that the experimental CD spectra for MoLox had a reduced signal compared to the predicted CD spectrum based on the crystal structure of MoLox (PDB: 4NRE [17]) (Figure S3). Overall, our data show that PmLox and TaLox have secondary structures that are congruent with the LOX fold.

Differential scanning calorimetry (DSC) was performed to examine the protein folding stability of these class II fungal LOXs. DSC provides complementary information to CD as both the melting temperature (T_m_) and the protein folding enthalpy, ΔH° (i.e., thermodynamics), can be obtained in a single DSC experiment. The thermodynamics parameters from the DSC experiments for the putative class II fungal LOXs are listed in Table 1. The T_m_ of MoLox determined from DSC, 62.5 ± 0.2 °C, is comparable to the T_m_ estimated previously from temperature-dependent CD spectroscopy [23]. The corresponding T_m_ values from the DSC thermograms of PmLox and TaLox are also similar to that of MoLox. The folding enthalpies, ΔH°, are notably higher for the putative class II fungal LOXs, relative to MoLox. Taken together with the CD data, these DSC results support that these class II proteins adopt a LOX-like fold.

### 3.3. Metal Analysis of Class II LOXs

Inductively coupled plasma-optical emission spectroscopy (ICP-OES) was used to analyze the metals present in the protein samples. Plant and animal LOXs contain a mononuclear, non-heme iron center whereas MoLox and other class I fungal LOXs contain a mononuclear manganese ion. The following four ions were selected for analysis: Fe, Mn, Cu, and Zn. It was previously seen in MoLox samples that low levels of contaminating Cu can be found and varied upon preparation [6,17,23]. ICP-OES performed on MoLox in this study revealed 0.74 atoms of Mn, compared to 0.13 atoms of Cu and low levels of Fe and Zn, per protein (Table S3). In our preparations, ICP-OES analysis of PmLox and TaLox samples showed equal parts manganese, copper and zinc. For comparison, ICP-OES was also collected for soybean lipoxygenase, a model iron LOX, resulting in 0.79 Fe atoms per SLO as expected.

### 3.4. Steady-State Kinetics of Class II LOXs

**Figure 4:**
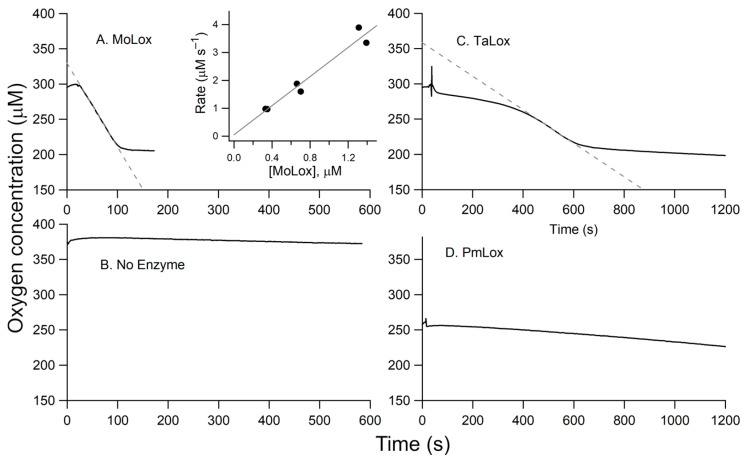
Representative Oxygraph traces. (**A**) MoLox (0.25 µM final enzyme concentration) reaction with LA. Panel (**B**) represents buffer only (no enzyme or substrate added). Additional raw traces correspond to the reactions of: (**C**) TaLox isolated from *P. pastoris* (5.0 µM), and (**D**) PmLox (3.52 µM). The inset in panel A presents the dependence of the observed rate of substrate oxidation versus enzyme concentration; these data were collected in duplicate. The gray dotted lines in panels (**A**,**C**) reflect the initial velocity measurements used to calculate enzyme rates. In all cases, the conditions were 0.1 M borate (pH 9) buffer, 100 µM LA, and 25 °C.

The primary structure of the initial oxidation product for the MoLox reaction with LA is a bis-allylic hydroperoxide, 11*S*-HpODE [6] (Scheme 1), which does not have a distinctive UV-visible spectrum. Thus, steady-state kinetic analysis of the MoLox reaction has been performed with an O_2_ electrode to track the consumption of the second substrate, molecular oxygen [23,24]. A representative Oxygraph trace for the reaction of MoLox with 100 µM of substrate LA is shown in Figure 4A. At all LA concentrations, the MoLox reaction exhibits a short lag phase (~20 s) upon enzyme addition (t = 0 s), followed by a linear decrease in O_2_ until the substrate is completely consumed (≤2 min), at which point the oxygen concentration plateaus (Figure 4A). At this 100 μM LA reaction, the rate was determined to be 2.7 ± 0.2 s^−1^ upon correcting the observed rate for the enzyme concentration. In the Figure 4A inset, we show the relationship of MoLox concentration on the reaction rates. The resulting linear relationship yields a slope with a value of 2.6 ± 0.3 s^−1^, which agrees well with the corrected reaction rate and the literature value for the MoLox *k*_cat_ with LA at this temperature [9,23]. Note that a Clark-type O_2_ electrode consumes a small amount of molecular oxygen during the electrochemistry experiment to monitor the concentration of oxygen dissolved in the buffer; as a result, there is typically a slow rate of oxygen consumption attributed to this background electrochemical monitoring. Figure 4B presents the background signal from buffer as a reference.

A representative Oxygraph for the *Ta*LOX reaction with 100 µM LA is presented in Figure 4C. Relative to the MoLox reaction (Figure 4A), there is a considerable lag phase for TaLox. This lag phase persists for several minutes compared to tens of seconds as seen for MoLox. Though a similar net change in O_2_ depletion is observed for both reactions, the TaLox reaction requires approximately five times longer to run to completion. Of note, the concentration of TaLox protein in the reaction cell was also nearly an order of magnitude higher than that of MoLox. Taken together, the TaLox reaction is associated with a slow rate of fatty acid oxidation, estimated at 0.04–0.05 s^−1^ after correcting for enzyme concentration.

Next, we examined the effect of pH and substrate. The first and second order rate constants for the MoLox reaction with LA was shown previously to be insensitive to pH from 7 to 9 [23]. In this report, steady-state kinetics were also collected for the TaLox reaction with LA at pH 7.0. The Oxygraph trace was noisy and had almost no apparent oxygen consumption. Due to the elevated critical micelle concentration (CMC) of fatty acids at pH 9, the remaining kinetic measurements were all performed at pH 9. Three common substrates were screened against TaLox for substrate specificity studies (Figure S4). TaLox demonstrated low, albeit non-zero LOX activity for LA and α-linolenic acid (ALA). The corresponding rates for ALA oxidation were approximately half as fast as for LA and the lag phase for ALA oxidation is nearly 3 times as long. The TaLox reaction with arachidonic acid (AA), a common fatty acid substrate for animal LOXs, did not show indications of oxidation, even over an hour reaction window. LA was therefore used for testing the kinetic activity of other class II fungal LOXs.

### 3.5. TaLox from E. coli Cultures

Next, we wanted to examine if the fungal LOXs might incorporate iron as the reactive metal. Attempts by our laboratory to enrich fungal LOXs with iron using *P. pastoris* and defined media were unsuccessful. A fungal LOX from *Fusarium oxysporum* was previously reported to bind iron as its reactive metal based on successful isolation from *E. coli* cultures [7]. Thus, we sub-cloned the TaLox gene into a bacterial vector and expressed and isolated the protein from *E. coli* cultures. Using the ferrozine assay, the protein purified in enriched media was found to contain 0.56 iron atoms per protein and is referred herein as ‘Fe-TaLox’. We also used protocols to substitute the Fe atom for Mn using biosynthetic approaches previously used by our laboratory for other LOXs [26]. With this approach, the Mn-TaLox form from *E. coli* was isolated with 0.81 Mn atoms per protein with minimal contributions from Fe, Cu, or Zn (Table S3) and is referred to herein as ‘Mn-TaLox’.

**Figure 5:**
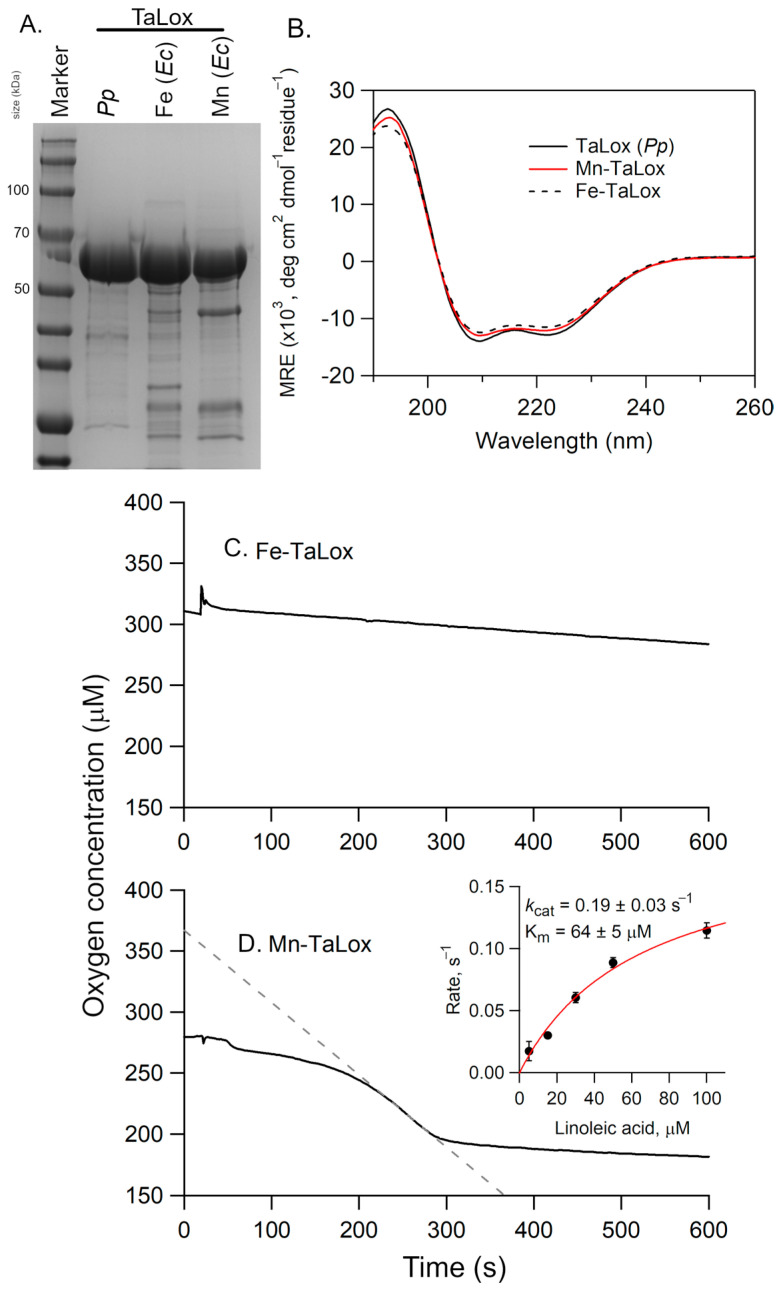
(**A**) SDS-PAGE analysis and (**B**) CD spectra of Fe- and Mn-TaLox isolated from *E. coli* (*Ec*) cultures and TaLox isolated from *P. pastoris* (*Pp*). In (**A**), the size of select molecular weight markers are shown along the side (in kDa). In (**B**), the CD spectra are normalized based on their protein concentrations and residue number (i.e., mean residue ellipticity, MRE). The buffer was 25 mM potassium phosphate, pH 7 and the temperature was 20 °C. Panels (**C**,**D**) present representative Oxygraph traces for the reactions of Fe- and Mn-TaLox, respectively, with LA. The enzyme concentrations were 3.24 and 5.0 µM, respectively. The inset represents the Michaelis-Menten plot; the rates, corrected for enzyme concentration, are the average of three independent measurements with the error bars representing ± s.d. In (**C**,**D**), the conditions were 0.1 M sodium borate (pH 9) buffer, 100 µM LA, and 25 °C.

All three forms of TaLox migrated at the same mass in the SDS-PAGE (Figure 5A). The CD spectra of these TaLOX samples from *E. coli* overlaid well with the CD spectra for the protein obtained from *P. pastoris* (Figure 5B). However, there was a difference in the DSC, with the Fe-TaLOX showing a reduced enthalpy of folding, ΔH°, compared to either Mn-TaLox or TaLox from *P. pastoris* (Table 1). Thus, the presence of manganese appears to impart a stabilizing effect to these putative fungal Lox structures.

The TaLox samples from *E. coli* were also kinetically analyzed using the O_2_ electrode (Figure 5C,D). The observed rate for fatty acid oxidation with Fe-TaLox was sluggish, with an activity, after correcting for enzyme concentration, of 0.01–0.03 s^−1^ at 100 µM LA. However, the Oxygraph of Mn-TaLox sample revealed notable oxygen consumption corresponding to an activity of ca. 0.12 s^−1^ at 100 μM LA. A Michaelis-Menten plot was generated with the substrate concentrations ranging from 5–100 µM LA (Figure 5D, inset). The resulting first-order rate constant, *k*_cat_, was determined to be 0.19 ± 0.03 s^−1^ and the Michaelis constant, K_m_, was 64 ± 5 µM. The former is about 15-fold lower than the MoLox reaction [9] and 50- to 100-fold lower than the GgLox reaction [11]. The K_m_ of the Mn-TaLox reaction with LA is elevated compared to that of MoLox (14 µM [9]) or GgLox (4.4 μM [4]).

### 3.6. SLO Leu Clamp Variants

Wild-type MoLox and TaLox display relatively low LOX activity, especially compared to plant and select mammalian isozymes [15,30,31]. Thus, to determine the functional consequence of a cysteine substitution at the Leu clamp position, class II-mimicking variants of the model plant LOX from soybean, SLO, were pursued. The leucine clamp (L546) of SLO was first mutated to a cysteine. For completeness, a second SLO variant, L546S (leucine to serine), was also prepared to assess the impact of introducing polarity at this residue. The two SLO variants, L546S and L546C, were purified as previously described from bacterial cultures (see ref [26]). Since plant LOXs are iron-containing, a ferrozine assay was performed. The ferrozine assay confirmed that both SLO variants bind iron to a high occupancy, with ratios of 0.89 and 0.80 iron atoms per protein (Table S4), respectfully. Of note, a ferrozine assay of wild-type SLO produced a value of 0.84 Fe atoms per protein, which agrees with the ICP-OES results described above. Thermodynamic analysis of the SLO protein variants indicated that L546C and L546S retained their folding stabilities relative to WT, though, most notably, SLO L546C has a slightly increased ΔH° and T_m_ value (Table 1). The latter observation was consistent with the elevated folding enthalpy seen for PmLox and Mn-containing TaLox relative to MoLox.

Steady-state kinetics were performed using UV-vis spectroscopy in single wavelength kinetics mode (λ = 234 nm) to follow the formation of the conjugated hydroperoxide, produced by SLO (Scheme 1). L546C and L546S were associated with a drastic decrease (~60-fold) in the first-order (*k*_cat_) and second-order (*k*_cat_/K_m_) rate constants, where *k*_cat_ represents the overall rate of the reaction and *k*_cat_/K_m_ value represents the efficiency of the enzyme reaction, including substrate capture. The three SLO Leu clamp variants, including the previously characterized L546A variant, display similar *k*_cat_ values. L546C has a notably lower *k*_cat_/K_m_ value than either the alanine or serine counterpart (Table 2). These data suggest that substrate capture may be less effective in the cysteine variant compared to the other Leu clamp variants.

To examine the impact of the cysteine on the enzyme mechanism, non-competitive kinetic isotope effects (KIEs) were performed using a deuterated substrate (D_31_-LA). The primary deuterium KIE on the first-order rate constant, ^D^*k*_cat_, was approximately 75, which is well within the range seen for wild-type and the L546X variants (Table 2). These values are in excess of the classical limit for transition state theory and fit with the hydrogen tunneling model that has been advanced for the SLO reaction [10,15,32]. The KIE on the second-order rate constant, ^D^*k*_cat_/K_m_, was associated with a large standard error for L546C, relative to the other variants, and attributed to a large uncertainty in the elevated values for the K_m_ (≥50 µM) parameter.

**Figure 6:**
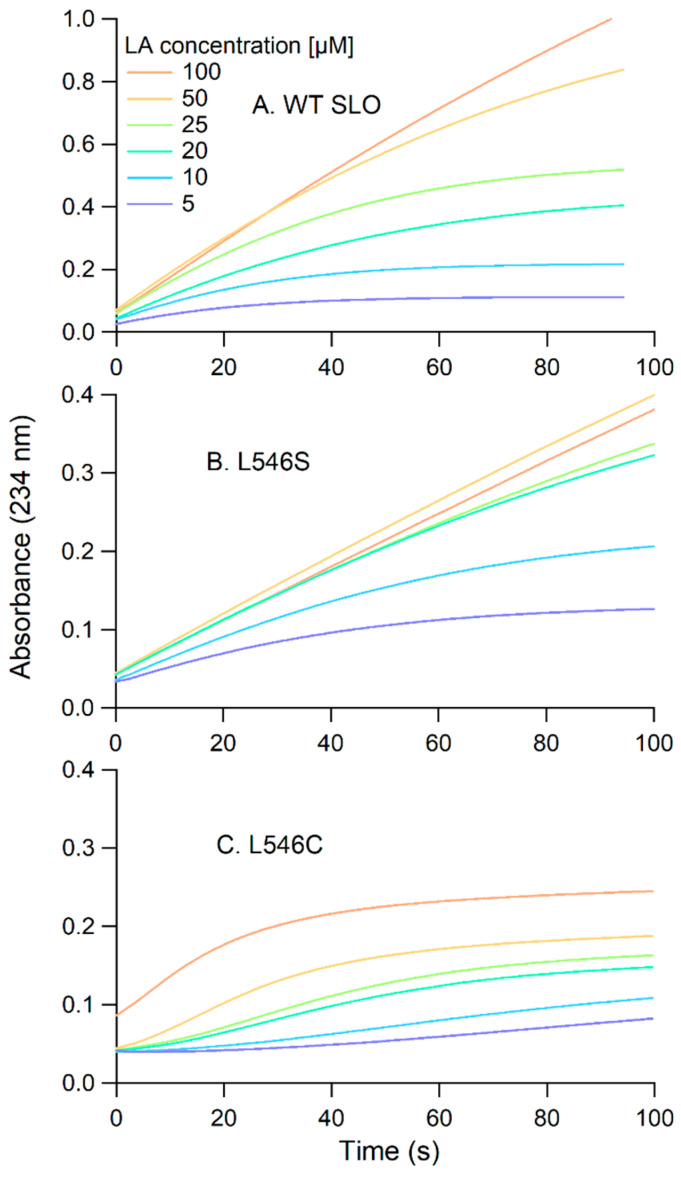
Representative UV-vis kinetic traces for the LA oxidation reactions with: (**A**) wild-type ([SLO] = 3.9 nM), (**B**) L546S (42.0 nM), and (**C**) L546C (70.5 nM) SLO. The buffer was 0.1 M borate, pH 9.0 and the temperature was 30 °C.

In addition to an inflated Michaelis constant, the raw kinetic traces for the L546C reaction revealed two distinguishing features (see Figure 6). First, L546C exhibited a lag phase notable at lower substrate concentrations. For example, at 5 µM LA concentration, the reaction showed at least a 30-s delay prior to the start of product formation. Note that this lag phase is dependent upon the substrate concentration. A lag phase was also observed for the reaction of TaLox with LA (Figure 4C). Second, L546C shows early abortion of turnover (Figure 6C); this indicates an inhibition effect with product formation and is not observed in reactions for other SLO variants (Figure 6A,B). For example, at the highest LA concentration of 100 µM, the L546C reaction plateaus at 0.2 Abs units (AU), whereas at 100 s for the L546S reaction, the Abs continues to increase beyond 0.4 AU. These data support that less than 50% of the total product is formed in L546C compared to that of WT or L546S. This effect could stem from the formation of the hydroperoxide product binding tightly to the enzyme complex, thus leading to either competitive or non-competitive inhibition of the enzyme.

To explore this possibility further, the SLO variants were pre-activated by substrate. SLO and other plant and animal LOXs are isolated in the resting state in the ferrous (Fe^2+^) oxidation state, which is converted to the active ferric (Fe^3+^) state during steady-state turnover. LOXs can also be activated by incubation of the resting state of the enzyme by aerobic incubation with two equivalents of substrate. Here, wild-type, L546S, and L546C SLO variants were activated aerobically by titrating the protein with LA and monitoring the rise in a broad feature centered at 330 nm in the UV-vis spectrum of the protein that corresponds to the emergence of the ferric species. These titration UV-vis spectra are displayed in Figure S5. Upon activation, all three proteins were dialyzed extensively against 0.1 M sodium borate buffer (pH 9) to remove any loosely bound substrate/product. Ferrozine assays revealed only slightly reduced levels of iron (0.6–0.65 Fe per SLO) after the activation.

Steady-state kinetics of the activated forms of WT and L546S produced similar rate constants relative to their pre-activated forms (Table S5), upon correction for the differing iron content in these variants. However, the activated form (Fe^3+^) of L546C exhibited a diminished activity, nearly 40-fold decreased compared to the pre-activated form. These kinetic analyses indicate significant inhibition of the activated form of L546C.

## 4. Discussion

In this report, we examined the kinetic and biochemical properties of three representative proteins that have been classified as a new sufamily of fungal LOXs. In these nine putative fungal LOXs, which are all annotated as manganese lipoxygenases in the NCBI, a cysteine is found in place of the leucine at the clamp position. We successfully expressed (recombinantly) and isolated these proteins using *P. pastoris* cultures. We found that these fungal proteins were rather poorly active as LOXs (*k*_cat_ ≤ 0.05 s^−1^).

Cysteine is a non-conservative mutation for leucine. In contrast to leucine, cysteine is a redox-active amino acid that undergoes oxidation in cells to initiate signaling pathways and to mitigate aberrant oxidation side reactions [33]. While Cys is well known to participate in the formation of disulfide covalent bridges with nearby cysteines, there are a broad range of cysteine post-translational modifications (see references [34,35,36], for examples).

CD spectra of the proteins revealed prominent α-helical content consistent with the LOX fold (cf. Figures S1 and S3). An unexpected result stemming from this study was that the predicted class II fungal LOXs presented more stabilized protein structures, based on elevated enthalpy of folding values (ΔH°), compared to MoLox. Both TaLox and PmLox, as isolated from yeast cultures, were determined with ΔH° values in the 1200 kJ/mol range, compared to ~800 kJ/mol for MoLox. Analytical SEC of PmLox and TaLox proteins showed elution profiles that are consistent with the expected monomeric forms of the protein (Figure S6); these data eliminate dimerization as responsible for the apparent differences in folding thermodynamics. It is important to note that a manganese-substituted form of SLO, an iron lipoxygenase, resulted in an inactive protein with a preserved protein structure that is slightly more stable than its native iron counterpart (Figure S7). The X-ray structures of both forms of SLO were nearly superimposable, with only subtle changes to the orientations of the metal-bound ligands [37]. Collectively, our thermodynamic analysis suggests that LOX protein stability may be dependent upon the nature of the bound metal in the active site, though the overall global structure is not significantly impacted.

SDS-PAGE analysis indicated that the putative class II fungal LOXs migrated at their theoretical masses and did not contain post-translational, *N*-linked glycosylation. MoLox, GgLox, and other class I fungal LOXs are all isolated with *N*-linked glycans (either from yeast cultures or from the native source). It is important to note that studies from our laboratory that demonstrated removal of the glycans from MoLox did not significantly impair activity or the global protein structure [24]. Thus, the minimal LOX activity observed from these annotated class II fungal LOXs is not attributed to the lack of post-translational modifications.

To gain some potential structural insight into the structural differences between class I and II fungal LOXs, AlphaFold-3 was used to build predicted structural models. The predicted models of the class II fungal LOXs align well with MoLox (Figure S1). All RMSD values were within 0.5 Å of the X-ray crystal structure of MoLox (PDB: 5FNO [17]). The RMSD values were reduced if the N-terminal segments that exhibit low pLDDT (<50) were omitted from the analysis. In an overlay of the active sites, the orientations of the primary metal-ligand residues of the class II LOXs are also nearly identical (Figure S1). Overall, AF3 was unable to provide clarity into the observed structural variations between MoLox and the class II LOXs.

The metal analysis of TaLox, as isolated from yeast, and PmLox showed an equal distribution of manganese, copper, and zinc binding. We wanted to explore if changing the metal was responsible for the reduced activity in these fungal proteins. Isolation of TaLox from *E. coli* cultures in enriched media resulted in 56% iron content, which is in range of some mammalian LOXs and select catalytically impairing LOX mutations [15,38]. Despite this, the activity of the Fe-containing TaLox protein was diminished compared to its yeast cognate. Further, the manganese form of TaLox from *E. coli* with M9-based minimal media showed elevated activity relative to the Fe-TaLox. The *k*_cat_ of Mn-TaLox was 0.19 ± 0.3 s^−1^ and correlates well to the activity of TaLox isolated from *P. pastoris*, based on the differences in the Mn content (ca. 81 vs. 33%). Thus, the residual LOX activity of TaLox likely stems from Mn as the cofactor.

Due to the sluggish activity of the fungal LOXs, the plant orthologue, SLO, was used as a proxy for determining the functional consequences of the non-conservative mutations to the leucine clamp in LOXs. The mutation of the Leu clamp of SLO, L546, to Cys revealed a detrimental impact to LOX function with three distinct features.

First, the SLO variant with the cysteine in the clamp location resulted in a more stable protein. The Leu residue does not bind to the metal, but the residue lies across from the Fe-OH bond and nestles substrate with respect to the metal center. The catalytic impairment of the mutations therefore does not arise from changes to the metal-ligand environment or metal content. The latter is consistent with the metal analysis, with both WT and L546C having similar iron concentrations. Further, CD spectroscopy shows no major differences in the secondary structure (Figure S8). The enhanced protein stability of the L546C variant, as determined by DSC (Table 2), is unlikely due to a large structural change in the protein. How this amino acid substitution results in a more stabilized structure is not understood, but the Leu-to-Cys substitution could alter the size of the substrate cavity and/or the amount of water inside the channel, thereby causing an increased stability of the protein structure. Similar behavior has been reported for RNase upon mutation of hydrophobic residues [39].

Second, in addition to the reduced *k*_cat_, the reaction of L546C with LA presents a kinetic lag phase. This could arise from either a sluggish binding of substrate and/or a slowed activation of the enzyme. For the latter, LOXs are isolated with the metal in the 2+ oxidation state that gets converted to the 3+ oxidation state upon aerobic incubation of the enzyme with substrate. Activation kinetics were not explored, but we show that the L546C protein can undergo activation under aerobic conditions, similar to that of WT or L546S variants. Consistent with the former, the K_m_ value of the L546C enzyme variant is elevated in comparison to WT SLO and other SLO clamp mutations (e.g., L546A/S). Importantly, an elevated K_m_ value was also noted for the Mn-TaLox reaction with LA (64 μM; Figure 5D inset). While K_m_ represents a complex ratio of microscopic rate constants for the substrate binding mechanism in SLO [14], a three-fold increase in K_m_ for L546C could be an indicator for weakened substrate binding and/or formation of a less productive enzyme-substrate complex.

Third, the reaction of the L546C exhibits early abortion. Since the K_m_ for LA is elevated for the L546C variant, competitive inhibition with the product seems unlikely. Alternatively, the hydroperoxide product, formed at the active site and adjacent to 546C, could react with the free thiol of the cysteine sidechain. This reaction could lead to the formation of a cysteine-hydroperoxide adduct or oxidation of the thiol [40,41], such as sulfenic or sulfonic acid. Consistent with this interpretation, the pre-activated (Fe^3+^) form of L546C was nearly inactive with LA. It was previously shown that the presence of free cysteine irreversibly inactivated LOX activity [42]. This is expected to occur through the redox reactions of cysteine with the hydroperoxide product to form oxidized cysteine side chains [33,40]. To examine cysteine oxidation further, we performed an Ellman’s test to quantify free cysteines for the resting and activated forms of the L546C variant of SLO. The Ellman’s test revealed a reduction in free cysteines from *ca*. 5.3 in the resting state to 3.7 following activation of the enzyme, compared to a theoretical five cysteine residues (Table S6). Taken together, our results implicate an oxidation of the active site cysteine at position 546 upon reactions with the substrate and/or product. These cysteine redox reactions lead to irreversible inactivation of SLO.

Kinetic isotope effects were pursued to investigate if the LOX mechanism changed upon mutation. KIEs of the SLO reaction revealed a large value for the first order rate constant, in line with that observed across all L546X variants, including WT (Table 2). The large ^D^*k*_cat_ is indicative of a non-classical, hydrogen tunneling mechanism for the transferred hydrogen atom [10]. A more sensitive kinetic property, which can be used to characterize the efficiency of hydrogen tunneling in these enzymes, is the magnitude of the temperature dependence of the KIE (i.e., ΔE_a_ = E_a_(D) − E_a_(H)) [10,14]. The reaction of the L546C variant is associated with a large K_m_, approaching the critical micelle concentration of the substrate (CMC = 130 μM [43]). This technical hurdle prevented the collection of precise *k*_cat_ and ^D^*k*_cat_ values across a temperature range needed to calculate activation energies. However, even with the increased uncertainty for ^D^*k*_cat_, the magnitude of this value is sufficiently large to allow us to conclude that the mechanism of hydrogen tunneling for C-H activation in LOXs is retained in the Leu-to-Cys mutation of SLO.

Our collective biochemical results presented herein raise the question, what is the function of these proteins, annotated as fungal LOXs, for the native host? Several annotated LOXs are also found in bacteria with unknown function [44]. Similar to these bacterial homologues, it is thus possible that the fungal proteins, annotated as LOXs, could have emerged as evolutionary relics. Further, these fungal proteins are predicted to be *N*-glycosylated and secreted [12], but over time a change in host preference might have led to a decreased need for secreted LOX activity. Alternatively, they may play a role in fatty acid transport or fatty acid-dependent oxidative stress by binding and neutralizing oxidized lipid products. While it could be a biochemically costly process for these proteins to serve as an antioxidant control of oxidized fatty acids, self-sacrificing enzymes, including oxidases, have been reported elsewhere [45,46,47,48]. It is notable that GgLox was the first fungal LOX to be discovered and was originally identified from analysis of host culture media [4]. Future work with culturing the native host coupled with transcriptional analysis, metabolomics, and/or knock-down study may help to resolve or confirm the function of these annotated class II LOXs.

## 5. Conclusions

In this report, we studied three representatives of a recently identified subclass of annotated fungal LOXs that present a natural substitution of the leucine clamp for a cysteine amino acid. Other than sharing some structural similarities to fungal LOXs, the collective biochemical data presented in this report suggests that these predicted class II fungal proteins are not effective LOXs. This was further supported by the poor and abortive enzymatic activity of the L546C variant of SLO. The native biological function(s) of these proteins that are annotated as fungal LOXs remain(s) unknown but may play a role in redox control and/or general fatty acid transport. These functions await experimental validation. The work further highlights the importance of the side chain bulk at the Leu clamp position in maintaining efficient LOX catalysis through a rate-limiting hydrogen tunneling mechanism.

## Figures and Tables

**Table 1:** Thermodynamic parameters of protein stability of class II LOXs determined by DSC ^a^.

LOX	ΔH° (kJ/mol)	T_m_ (°C)
MoLox	768 ± 10	62.5 ± 0.2
PmLox	1239 ± 21	60.1 + 0.2
TaLox (*P. pastoris*)	1242 ± 4	59.8 ± 0.4
Fe-TaLox (*E. coli*)	729 ± 16	57.6 ± 0.1
Mn-TaLox (*E. coli*)	1092 ± 4	57.1 ± 0.2
WT SLO	1471 ± 24	62.6 ± 0.3
L546C SLO	1646 ± 6	64.7 ± 0.3
L546S SLO	1291 ± 14	62.7 ± 0.2

^a^ The data represent the average ± s.d. from two separate experiments. The buffer for the fungal LOXs was 50 mM HEPES, 150 mM NaCl, pH 7.5, while the buffer for SLO was 100 mM sodium borate, pH 9.0.

**Table 2:** Kinetic parameters of SLO Leu clamp variants *^a^*.

SLO Variant	*k*_cat_ (s^−1^)	*k*_cat_/K_m_ (s^−1^ μM^−1^)	^D^ *k*_cat_	^D^*k*_cat_/K_m_
WT	288 ± 23	10.7 ± 1.2	64 ± 3	42 ± 4
L546A *^b^*	4.8 ± 0.6	0.33 ± 0.1	93 ± 9	N.R. *^c^*
L546C	5.2 ± 0.6	0.06 ± 0.01 *^d^*	75 ± 15	71 ± 34 *^d^*
L546S	3.9 ± 0.1	0.64 ± 0.04	109 ± 6	98 ± 10

*^a^* Reactions were carried out with LA as substrate in 0.1 M sodium borate (pH 9.0) buffer. Rate constants are reported at 30 °C. The data represent average ± standard error from at least triplicate data set. *^b^* L546A data from reference [15]. *^c^* N.R., not reported. *^d^* Large uncertainty for K_m_ values.

## Data Availability

The data presented in this study are available upon request.

## Supplementary Materials

The following supporting information can be downloaded at: https://www.mdpi.com/article/10.3390/biom15081153/s1, Figure S1: AlphaFold-3 predicted structures of class II fungal LOXs; Figure S2: SDS-PAGE of MoLox and TaLox; Figure S3: Predicted CD spectra of fungal LOXs; Figure S4: Representative Oxygraph traces for the reactions of TaLox with various fatty acid substrates; Figure S5: UV-visible spectral titrations for the aerobic activation of SLO by LA; Figure S6: FPLC-SEC traces of fungal LOXs; Figure S7: Baseline-corrected DSC thermograms for Fe- and Mn-forms of WT SLO; Figure S8: CD spectra of SLO variants; Table S1: Summary of properties of fungal LOXs; Table S2: Sequences of predicted class II fungal LOXs; Table S3: ICP-OES metal analysis of SLO and fungal LOXs; Table S4: Ferrozine assay for iron content in LOXs; Table S5: Kinetics of pre- and post-activated forms of SLO; Table S6: Ellman’s test results for L546C.

## Abbreviations

The following abbreviations are used in this manuscript:

LOX	Lipoxygenase
DSC	Differential scanning calorimetry
CD	Circular dichroism
KIE	Kinetic isotope effect
WT	Wild-type
SLO	Soybean lipoxygenase
LA	Linoleic acid
SDS-PAGE	Sodium dodecyl sulfate polyacrylamide gel electrophoresis
