# Supplementary material for: Biochemical Consequences of a Leucine-to-Cysteine Clamp Substitution in Lipoxygenases
Source: Biomolecules. 2025 Aug 11;15(8):1153. doi: 10.3390/biom15081153 (PMC12383934; doi:10.3390/biom15081153)
Supplement: Supplementary file 1 [file biomolecules-15-01153-s001.zip › biomolecules-3728712-supplementary.pdf]

Supporting Information

Biochemical Consequences of a Leucine-to-Cysteine Clamp Substitution in Lipoxygenases

S. Gage Hill, Katherine DeFeo, Adam R. Offenbacher *

Department of Chemistry, East Carolina University, Greenville NC 27858 USA

* Email: Offenbachera17@ecu.edu

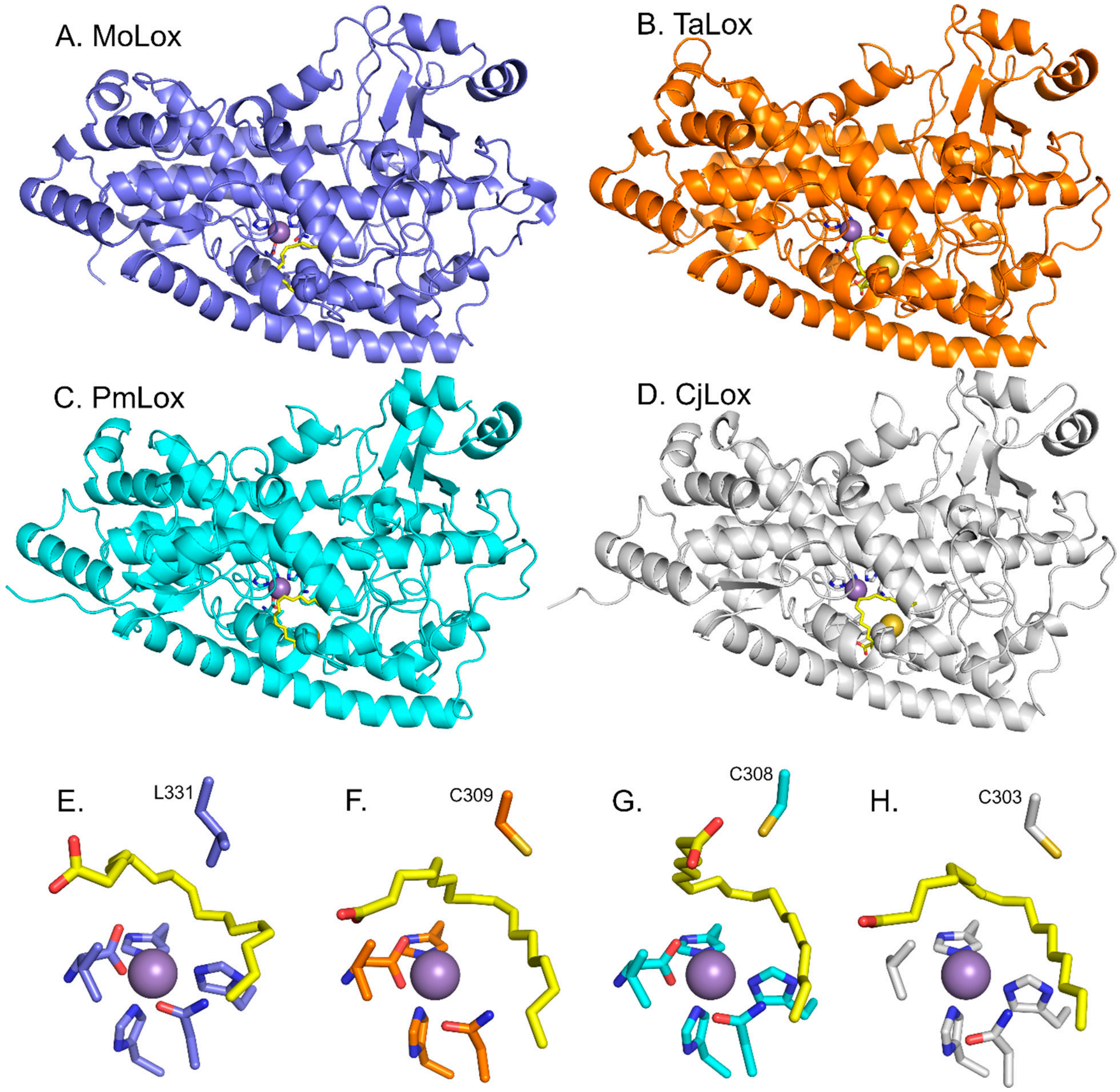

Figure S1. AlphaFold3 predicted structures of class II fungal LOXs. Panels A-D show the global structures of (**A**,**E**) MoLox (PDB: 5FNO) (**B**,**F**) TaLox (ipTM = 0.93, pTM = 0.94), (**C**,**G**) PmLox (ipTM = 0.95, pTM = 0.95), and (**D**,**H**) CjLox (ipTM = 0.93, pTM = 0.95). The Leu clamp and cysteine substitutions are shown as spheres. The manganese center is depicted as a purple sphere. A fatty acid is modeled into the active site (shown as yellow sticks). Corresponding active site structures are depicted in (**E**−**H**). The amino acid numbering for the Leu or Cys clamp residue is shown for reference.

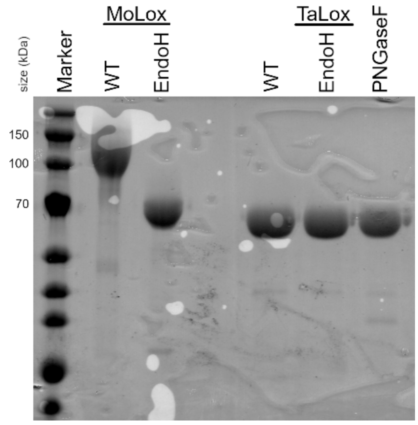

Figure S2. SDS-PAGE of MoLox and TaLox. Samples were also treated with EndoH and PNGase F to remove glycans.

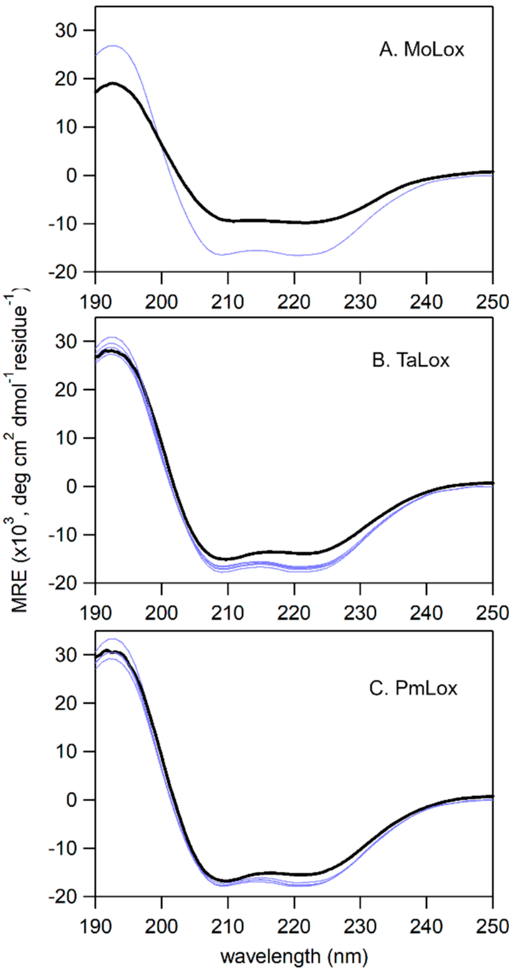

Figure S3. Predicted CD spectra (light blue traces) of fungal LOXs. The experimental CD data (in mean residual ellipticity) are represented as bold black lines and represent: (**A**) MoLox, (**B**) TaLox, and (**C**) PmLox. The predicted CD spectra were calculated using PDBMD2CD online server. The predicted CD spectra for PmLox and TaLox were generated using the five generated AF3 predicted models.

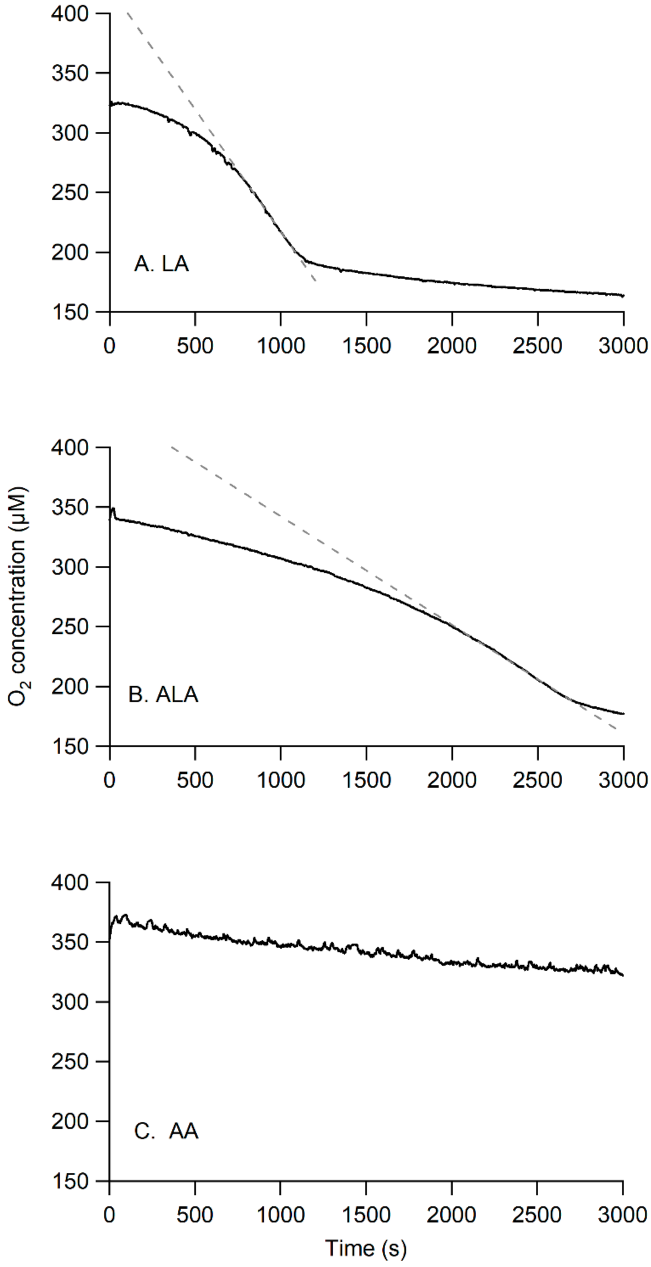

Figure S4. Representative Oxygraph traces for the reactions of TaLox (*P. pastoris*, 4.5 µM) with various fatty acid substrates: (**A**) linoleic acid, LA; (**B**) α-linolenic acid, ALA; (**C**) arachidonic acid, AA. The buffer was 0.1 M sodium borate, pH 9.0, substrate concentration was 100 µM, and 25°C.

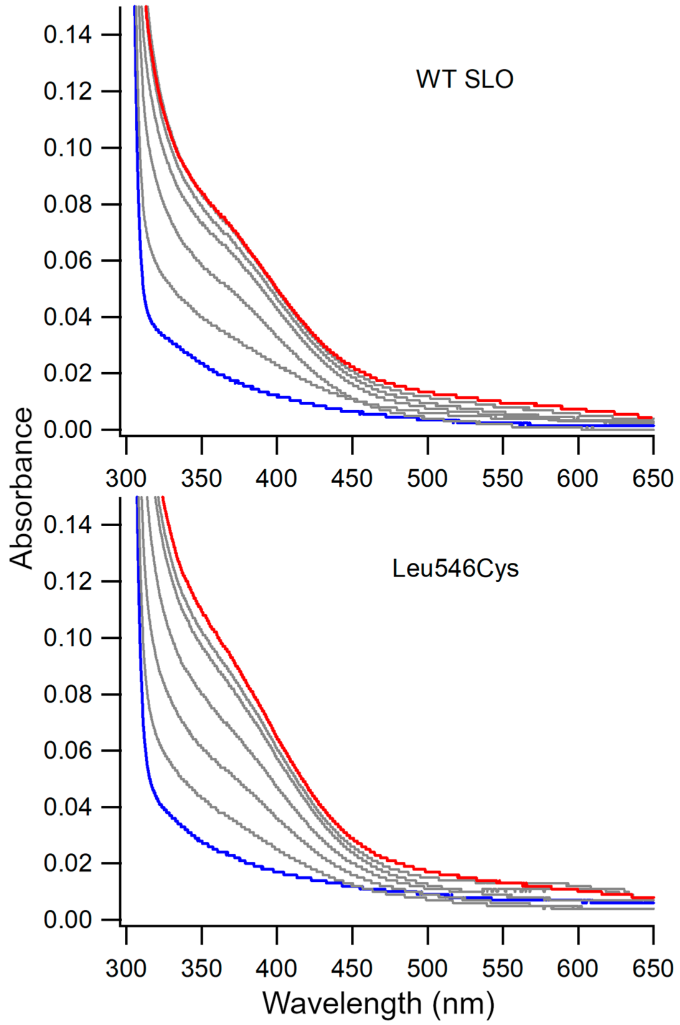

Figure S5. UV-visible spectral titrations for the aerobic activation of SLO by LA. The resting (Fe^{2+}) and activated (Fe^{3+}) states are depicted by the blue and red traces, respectively.

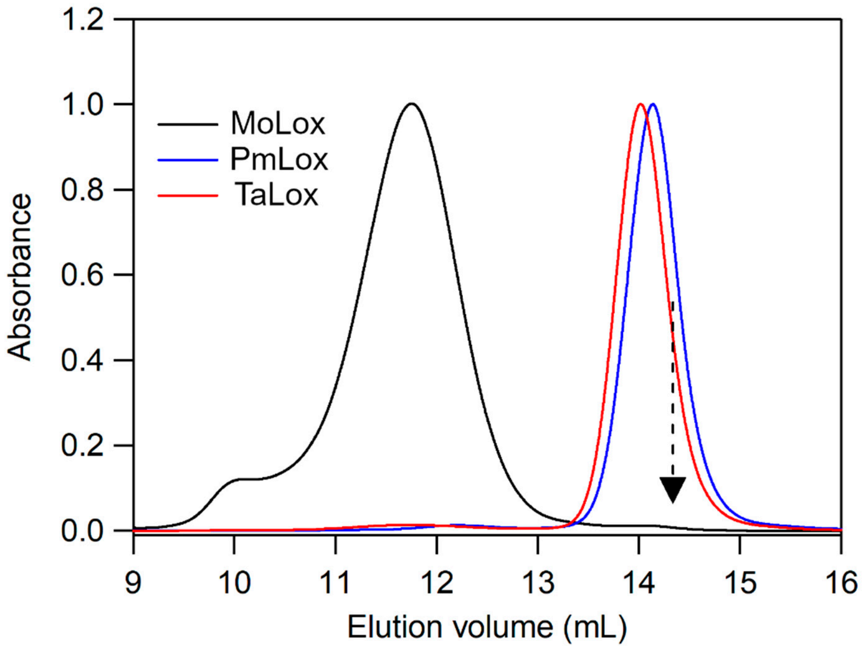

Figure S6. FPLC-SEC traces of fungal LOXs. MoLox, PmLox, and TaLox were eluted using a Superdex^{TM} 200 Increase 10/300 GL analytical column that was pre-equilibrated with 50 mM HEPES (pH 7.0), 150 mM. The elution migration of bovine serum albumin (66 kDa) is displayed as an arrow for reference. The absorbance maxima were normalized for clarity.

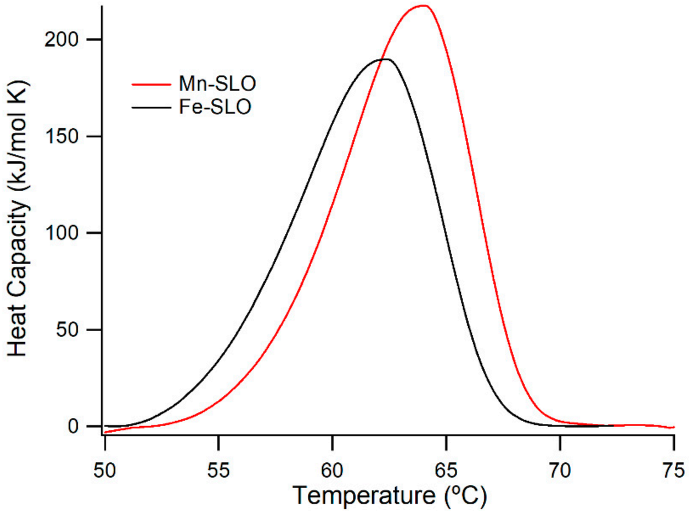

Figure S7. Baseline-corrected DSC thermograms for Fe- and Mn- forms of WT SLO. The buffer was 0.1 M sodium borate, pH 9.0 and the scan rate was 1 ºC/min.

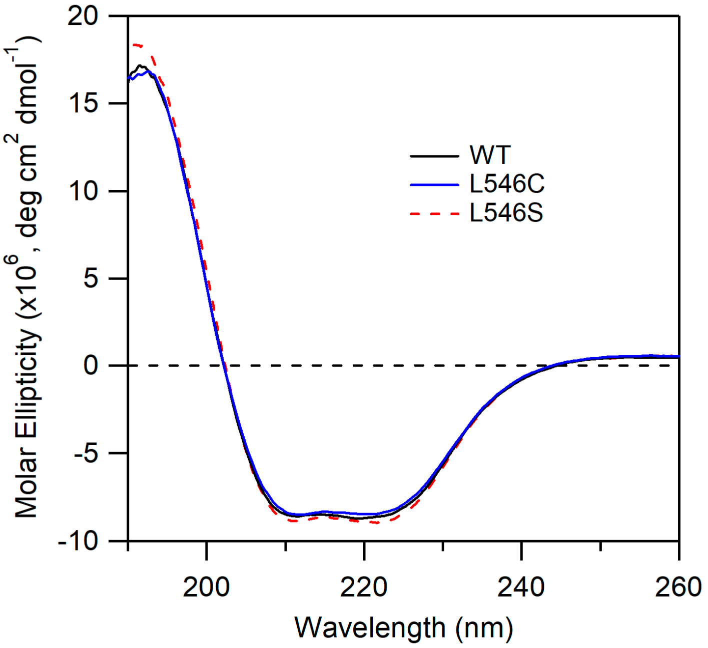

Figure S8. CD spectra of SLO variants. The buffer was 0.1 M sodium borate, pH 9, and the temperature was 20 °C.

Table S1. Summary of properties of fungal LOXs.

Enzymes	Length	Theoretical Mass (kDa)	Predicted Glycans*	Percent Identity
Magnaporthe oryzae, MoLox	604	67.0	7	100%
Trichoderma arundinaceum, TaLox	585	65.0	2	42%
Periconia macrospinosa, PmLox	595	66.6	7	39%
Cordyceps javanica, CjLox	575	63.9	2	40%

* using the NetNGlyc 1.0 server

Table S2. Sequences of predicted class II fungal LOXs

Fungal LOX	Protein sequence (secretion sequence is underlined)
CjLox<br>(TQV99024)	**MRTTLATALLCMLLDGASA**APQPRDAAVCIPQRDSNPRGRAARVSERNSGFV<br>YGPSLIGEAAPFPNGTLGNARSKADYALWSVDRKEIDGRIAADLQQIQASVEAN<br>GGLKTWDDYGRILYDGQLKQSNPRGPAPGIIANATQDLLFSMERLSEHPYAVRL<br>VAATERLPFNVDAKVVGKLVGAGITLHSLQASKSLFLVDHSYQKQYTLPSVVT<br>RYPVACSAYFYIDPKTKNFLPLAIKTNSGSDLIYTPLDSPEDWLLAKMMFNAND<br>MFHAQMLHLVISHDVSESVHQAALHTLSDNHPVMVILERLMLQGYSSRIVGEE<br>LCFNPGGHWDQLMAYDQFSCRKFVSDQWPVSGKFQAGYFETDLKSRGLLNDH<br>GVSVFKSFPFWDDAKEIRDAYKAFFKSFVDSYYKSELDLAGDFELQNWFAEAS<br>EHAKTQDFPSKHSLSKGTLVDVLAHFGFILSVGHHSMNGGAPIASGTLPFHIPAL<br>YTAPPTAKGIKDLLPYLPDVPTALHYLGFMASFNRPFYPSDGRTLEKAFSEQGM<br>LNKLNQATNDAAAQFLKSLQILSTKIQARKFDNNGLSQGMPFIYRTLDPNYIPFF<br>CAV
PmLox<br>(PVH93852)	**MPRLALMSLLLACSTSVTAAA**LHIRQSDSNGTTTVTIPQRDADRAARKKEVA<br>YRHDNFLYNISQIGNAAAFPMGKIGEERVSAAWDQWQIDRDIINGHIQKDVAQ<br>IRQAIVANNGTLRTLDDYATVLYKDQWLNASPLKPALGSLTNYTLDSFFGGER<br>LVRPYSLYKASDKDSKLIDISDEDAKKIAGSTVAELLSANRLFAVDHSYQADQ<br>TVYVPSQFNDKYGAPVTALFYLNDNKELLPLGIRTNTGANLTYTPLDGENDW<br>LLAKMMFNVADQFHAQIYHLTATHNVGEALHEAAMRTLSDAHPIMAVLDRL<br>NYQAYSARPVGEAMCFNPMGHWDENFHISQIGCRNFVTEYWPTYGAFEPNYL<br>QTDLHARGLVDEAGISPFKTFPFWDDASEILRVQREFFTTFVDTYYTSDEEVTA<br>DEEVGDWFEEVRRGPTGPEVEAQGLTPVASFPEKAAKKVLVDVLTHNAWLQ<br>VAHHSLNAGDPVRSSLTLPFHPGGLHKPVPEAKGIESVVPFLPNATASVTSIGF<br>SASFNRPRYRTMDPPRTLAYAYSGPEFLAHFAEKEVHDAADKYLEDMTRLGE<br>KNDARKIEADGMCTGQGIPFCWTAINPSYIPWFFSV
TaLox<br>(RFU77717)	**MKAQAACLLLNSLALVAEA**APHQWQAVARQGVSQASLPQNDRDRKARAAE<br>VAARNVGFVYGPSLIGEAAPFPNGTLGNALTQSDMDLWSVDRDDIDSRIMSDV<br>AAIKPAIAANGGLKSLDDYANVLYQGQWKNANPRGVAPGIMTNYTQDLLFSM<br>ERLSQNPYSLVFLKPSDELPFALDDDTTRKIAGATLEELHASSSLFFVDYSWQSE<br>LPKTTVAPQRYGAASSAYFYLHPESKDFLPLAIKTNAGKDLIYTPLDSDNDWLL<br>AKMIFNVNDLFHSQMLHLVITHDVSEAVHEAALHTLSPKHPVMVILDRLMLQA<br>YSSRIVGEELCFNPGGHWDQLMYINNDGCRDYVTQTWPTSGRFQAGYLHTDLK<br>ARGLINDQGEYPFKAFPFFQDASEIHDAYRAFFQSFVDSYYASDKDVVADWEV<br>QSWFIEATARAQVQDFPKASPASPVTKDTLVDVLTHFGFIVSVGHHALNGGDP<br>VGSKATLPFHLPALYAPLPEAKGVTDLIPFLPPAANAIQYIGFIASFNRPFYETSD<br>RTLKAAFSEDSMLAKLNKETNDAASEFVNSMQDLSNRVRSRGFDENGLSMGM<br>PFVYRTLDPNYIPFFCAV

*Genbank numbers are in the parantheses

Table S3. ICP-OES metal analysis of SLO and fungal LOXs.

Enzyme	Fe:LOX	Mn:LOX	Cu:LOX	Zn:LOX
SLO	0.79	0.05	0.04	0.01
MoLox	0.02	0.74	0.13	0.06
TaLox (*P. pastoris*)	0.01	0.39	0.31	0.22
PmLox	0.01	0.32	0.31	0.27
Mn-TaLox (*E. coli*)	0.04	0.81	0.04	0.05

Table S4. Ferrozine assay for iron content in LOXs.

LOX Sample	Fe:LOX
SLO	0.84
SLO 546S	0.89
SLO 546C	0.80

Table S5. Kinetics of pre- and post-activated forms of SLO [a.]

	Resting state, Fe^{2+}		Activated state, Fe^{3+}	
SLO variant	k_{cat} (s^{-1})	K_m (μM)	k_{cat} (s^{-1})	K_m (μM)
WT	288 ± 23	26 ± 2	286 ± 8	11 ± 1
L546S	3.9 ± 0.1	6.1 ± 0.4	2.95 ± 0.07	4.0 ± 0.5
L546C	5.2 ± 0.6	86 ± 15	0.13 [b]	N.D. [c]

[a] Rates are corrected for Fe content. [b] Estimated value based on the rate at 100 μM LA. [c] N.D., not determined.

Table S6. Ellman's test results for L546C.

SLO	Expected free Cys	Quantified for Fe^{2+}	Quantified for Fe^{3+}
L546C	5	5.1–5.4	3.6–3.8
